# Applying a systems perspective to understand the mechanisms of the European School Fruit and Vegetable Scheme

**DOI:** 10.1093/eurpub/ckac054

**Published:** 2022-11-29

**Authors:** Mahshid Zolfaghari, Biljana Meshkovska, Anna Banik, Carlijn B M Kamphuis, Birgit Kopainsky, Aleksandra Luszczynska, Celine Murrin, Nanna Lien

**Affiliations:** Department of Nutrition, University of Oslo, Oslo, Norway; Department of Nutrition, University of Oslo, Oslo, Norway; Wroclaw Faculty of Psychology, SWPS University of Social Sciences and Humanities, Wroclaw, Poland; Department of Interdisciplinary Social Science, Utrecht University, Utrecht, The Netherlands; Department of Geography, System Dynamics Group, University of Bergen, Bergen, Norway; Wroclaw Faculty of Psychology, SWPS University of Social Sciences and Humanities, Wroclaw, Poland; School of Public Health, Physiotherapy and Sport Science, University College Dublin, Dublin, Ireland; Department of Nutrition, University of Oslo, Oslo, Norway

## Abstract

**Background:**

For the past two decades, the percentage of European children who consume fruit daily has remained at around 40%, despite numerous school-based policy efforts and interventions. This study aimed to apply a systems approach to provide an integrated perspective of the mechanisms of the European School Fruit and Vegetable Scheme (the Scheme) to understand better how to increase its long-term impact on children’s fruit and vegetable consumption.

**Methods:**

We developed a causal loop diagram by synthesizing peer-reviewed articles and national government documents related to the Scheme, following the conceptualization steps of system dynamics. The initial causal loop diagrams were then validated in three stages by consulting with experts (two individuals and a group) in school-based fruit and vegetable programmes, children's fruit and vegetable consumption and the Scheme, using disconfirmatory interview guidelines.

**Results:**

The findings suggest that a central self-reinforcing mechanism through which children socialize during fruit and vegetable consumption is critical in the habituation process. Additionally, the initial increase in children’s fruit and vegetable consumption following the Scheme implementation is due to growth in three self-reinforcing loops related to motivation and capability mechanisms; however, this trend gradually slows and stops due to four balancing feedback loops with alternative goals related to opportunity mechanisms that reach their limits.

**Conclusions:**

The scheme's design should incorporate activities that align the objectives of the implementers and recipients of the Scheme at all levels. This alignment should provide children with ongoing opportunities to consume fruits and vegetables and strengthen the motivation and capability mechanisms.

## Introduction

Establishing healthy eating habits early and maintaining them throughout life is a key policy objective for preventing the early onset of non-communicable diseases (NCDs) and obesity.^1^ Since the late 1990s, a key recommendation for healthy eating has been to have five portions a day of fruits and vegetables (FV).^2^

School-based FV multi-component intervention studies and policies have been the primary strategy to reach children.^3^ However, although the proportion of daily consumers of fruit varies by country, age, gender and socioeconomic status (SES), on average only 40% of European 7–9-year-olds^4^ and 11–13–15-year-olds^5^ consume fruit daily and there has been little change since 2000.^6^

Against this background, it is relevant to reconsider the school-based FV policies to identify which mechanisms are at work to achieve a long-term impact on children’s FV consumption. The European School Fruit and Vegetable Scheme (the Scheme), implemented in 26 member states for more than 10 years, serves as a useful case study for illuminating mechanisms and proposing strategies that utilize them to maximize the Scheme’s impact.

The Scheme has been implemented under the European Commission (EC) Common Agricultural Policy since 2009/2010. Its primary objectives are to increase children’s FV consumption, stabilize European FV markets and decrease childhood obesity.^7^ The Scheme aims to achieve its objectives through three components: direct FV provision, educational measures and information measures. The EC contributes up to 150 million euros per year to the Scheme’s FV part, with funds allocated based on each member state’s child population and regional development.^8^ The design and implementation of the Scheme are left to the participating countries. However, they are required by the EC to develop a 6-year country strategy when applying to participate. The research findings on the Scheme’s short-term impact (i.e. 12–18 month follow-up assessment) on children’s FV consumption^9^^,^^10^ are consistent with a meta-analysis of similar school-based policies that found a mean effect of 0.28 portions per day, primarily on fruit consumption.^3^ There is little information available about the Scheme’s long-term impact. However, a 6-year follow-up study in Irish schools found that children who liked the Scheme continued to eat three or more portions of FV every day,^11^ which is consistent with findings from a similar Norwegian FV programme.^12^ In the latter, children received free FV for an entire school year, and effects were found after 3^12^ and 7 years,^13^ but not after 14 years.^14^ In Ireland, the Scheme is built around a multi-component FV promotion intervention^15^ that spans only 16 days, whereas, throughout the rest of the member states, the main component is FV provision, which in some countries can last the entire school year.^8^ Other studies of the Scheme’s effectiveness have addressed the Scheme’s intensity,^16^ the social environment, like teacher training^17^ and the educational measures,^18^ as well as the role of parental involvement^15^ and FV suppliers’ participation.^19^ These provide in-depth knowledge of a specific component but do not enable us to see the inter-relationships between these agents and components.

The Scheme or similar school-based FV programmes are comprised of various people, institutions, sectors and factors that interact and influence the potential long-term impact on children’s FV consumption. Systems thinking and system dynamics approach provide alternative ways to assess impact.^20^ This article provides an integrated perspective of the multiple settings, agents, components and interconnected mechanisms that are at work in this nutrition policy programme to achieve a long-term impact on children’s FV consumption and identify ways to enhance their influence.

## Methods

**Figure 1:**
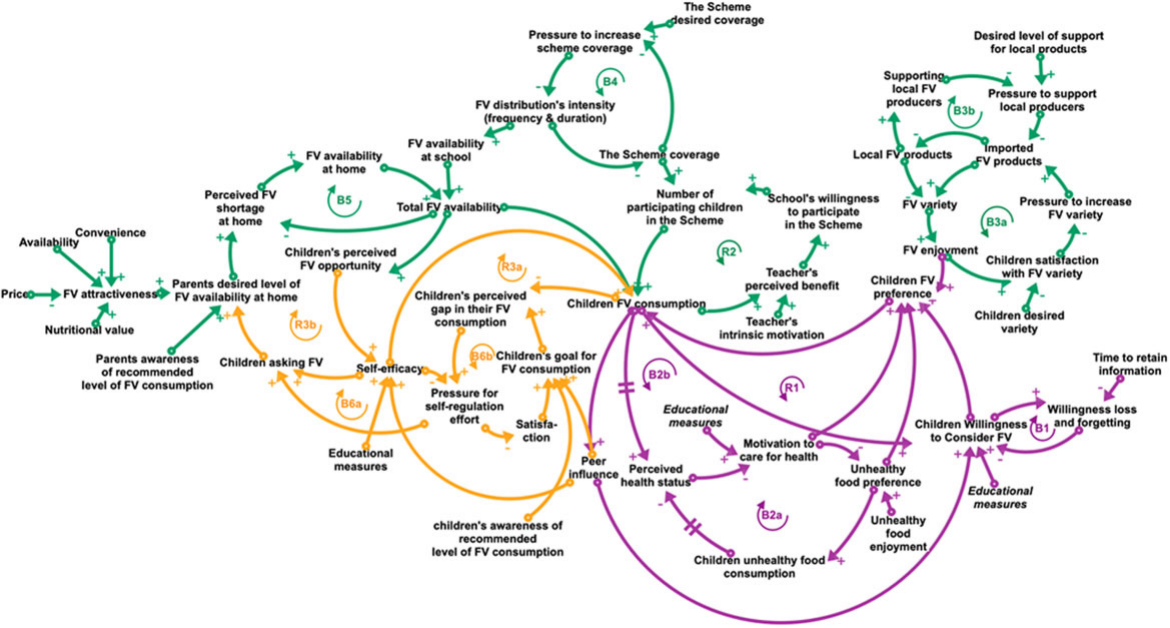
A complete view of the causal loop diagram (CLD) illustrating the motivation (purple), opportunity (green) and capability (orange) mechanisms regarded as important in influencing children's FV consumption in the Scheme. A CLD visualizes the interconnections among variables as well as feedback loops (FBL). For each arrow, the polarity indicates whether variables move in the same (positive sign) or opposite (negative sign) directions (all else being constant). There are two types of FBLs in a CLD: balancing FBLs (B), which oppose change introduced to the system, and reinforcing FBLs (R), which amplify change introduced to the system

We applied systems thinking and the conceptualization steps from the system dynamics approach^20^ and used peer-reviewed articles and official documents to identify and represent the interconnected mechanisms at work in the Scheme to achieve a long-term impact on children’s FV consumption. Systems thinking uses causal loop diagrams (CLD) to depict the feedback structure of systems and to capture and communicate hypotheses about the causes underlying the behaviour over time in a system.^20^ A CLD visualizes the interconnections among variables as well as feedback loops (FBL), as shown in figure 1. For each arrow, the polarity indicates whether variables move in the same (positive sign) or opposite (negative sign) directions (all else being constant). There are two types of FBLs in a CLD: balancing FBL (B), which oppose change introduced to the system, and reinforcing FBL (R), which amplify change introduced to the system.^20^ It is important to note that the CLD presented in this article is based on evidence that varies in strength and methodology; thus, it represents a hypothesized causal association rather than direct causality.

**Figure 2:**
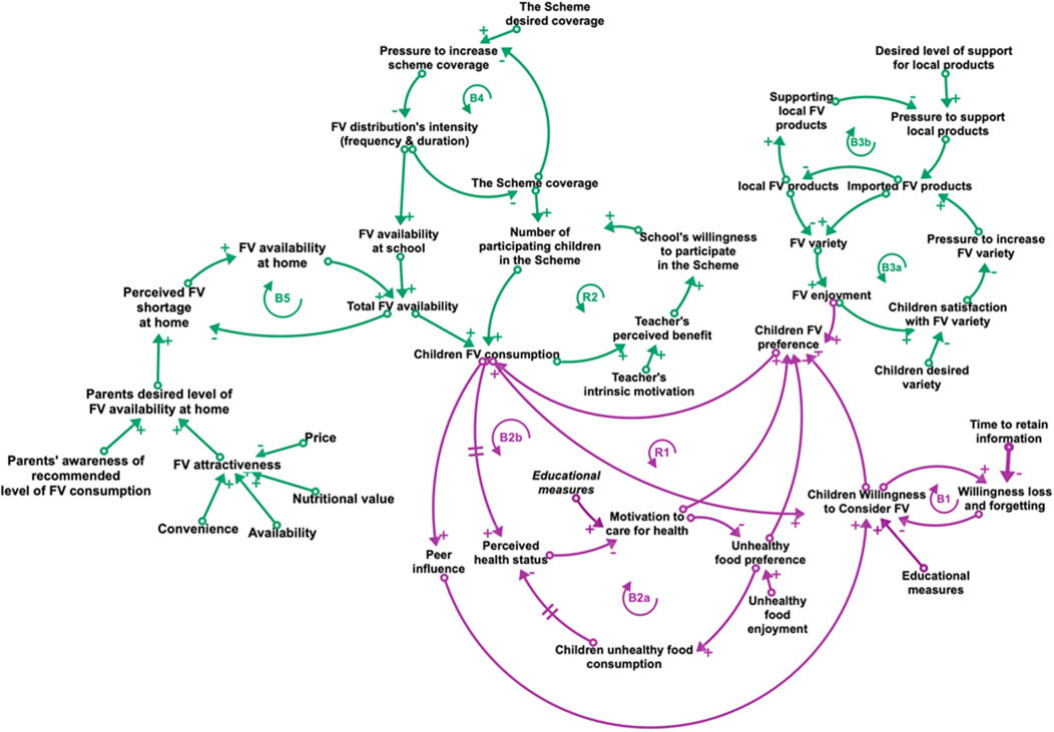
A causal loop diagram (CLD) illustrating motivation (purple) and opportunity (green) mechanisms considered as important in influencing children's FV consumption in the Scheme. A CLD visualizes the interconnections among variables as well as feedback loops (FBL). For each arrow, the polarity indicates whether variables move in the same (positive sign) or opposite (negative sign) directions (all else being constant). There are two types of FBLs in a CLD: balancing FBLs (B), which oppose change introduced to the system, and reinforcing FBLs (R), which amplify change introduced to the system

**Figure 3:**
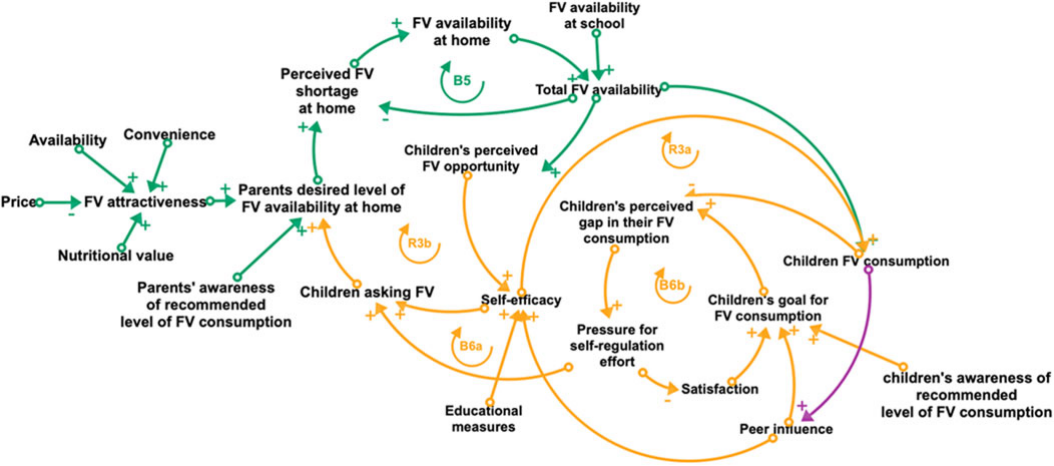
A causal loop diagram (CLD) illustrating capability mechanisms (orange) viewed as important in influencing children's fruit and vegetable (FV) consumption in the Scheme. A CLD visualizes the interconnections among variables as well as feedback loops (FBL). For each arrow, the polarity indicates whether variables move in the same (positive sign) or opposite (negative sign) directions (all else being constant). There are two types of FBLs in a CLD: balancing FBLs (B), which oppose change introduced to the system and reinforcing FBLs (R), which amplify change introduced to the system

Our research is based on publicly available data and, as such, did not necessitate approval from the institutional ethics committee.

### Data collection

We drew on documents related to the Scheme, which were gathered from the EC’s website and an EC policy officer responsible for the Scheme. These documents, together with peer-reviewed studies on the Scheme, enabled initial comprehension of it, including its main structure, success variables and features related to its effectiveness. In addition, we searched for scientific articles on system dynamics or systems thinking that addressed the same problem to make sure we built upon the existing evidence. Finally, peer-reviewed articles on the determinants of children’s FV consumption and school-based FV programmes were gathered to further develop and strengthen the initial CLD with empirical evidence. Gathering research articles and grey literature involved searches of scholarly databases (e.g. PubMed, Web of Science and Google Scholar) and institutional websites.^8^^,^^21^ In the final CLD, 39 studies were included (see Supplementary material S1, where a complete list of the literature used in developing the CLD is provided).

### Developing the CLD

Five key steps for developing a CLD from raw qualitative data^22^ were followed. Step 1 began by open coding, which included reading every document on a line-by-line basis to identify statements about cause-and-effect relationships that pertain to the Scheme’s impact. The output of this step was a list of data segments that varied in length from one sentence to one paragraph. Step 2 entailed extracting variables and their relationships from the identified data segments; as a result, we re-read the data segments from Step 1 and deconstructed each argument to cause and effect and their relationship. Step 3 entailed transforming texts into words-and-arrow diagrams; hence, the cause-and-effect relationships found in step two were converted to a causal diagram consistent with system dynamics language. Step 4 was axial coding, which involved reassembling the words-and-arrow diagrams by identifying relationships between variables to combine them into a single map. Step 5 ensured that the CLD remained connected to the original data source. Hence, we documented each link with its relevant data segment in an excel spreadsheet (see Supplementary material S2, where an example is included to demonstrate how to create a CLD from textual data). A complete list of inter-relationships in the CLD, exemplary data segments and corresponding references, is available upon reasonable request from the first author.

### Validating the CLD

We presented the CLD to experts in three 1-h online meetings. The experts included an economist previously involved in the Scheme evaluation, a public health nutritionist who had worked on establishing school-based FV programmes, and a group of eight public health nutrition researchers with expertise in children’s FV consumption and school-based interventions. The presentations were conducted in alignment with the disconfirmatory interview guidelines.^23^ Following a brief introduction to the system dynamics approach, we verbally described the structure and behaviour of each FBL. The experts had the opportunity to express their concerns and questions about the CLD representations both during and after the presentations. The determinants of FV consumption, the Scheme’s impact on developing FV consumption habits, and the effort required to implement the Scheme in schools were some of the main points of the discussions. The first two points led to further literature search and analysis, and we modified the CLD accordingly. The latter was not pursued because it was outside the scope of this study. The core research team (M.Z., B.M. and N.L.) met weekly to revise the CLD and evaluate the key variables and their causal relationships. In case of any disagreement, more literature to deepen our understanding was gathered, and analysis resumed. Additionally, the CLD was validated by an independent reference group with comparable skills to the experts above (A.B., C.K., B.K., A.L. and C.M.). A 2-h workshop was held to address the co-authors’ concerns, and one further link was added to the CLD. Additionally, see Supplementary material S3, which contains additional information about the method.

## Results

Figure 1 depicts an overview of the CLD, demonstrating how the Scheme’s interconnected mechanisms are at work to achieve a long-term impact on children’s FV consumption. The CLD was sorted into three overarching areas: motivation, opportunity and capability.^24^

### Motivation

Motivational mechanisms for children’s FV consumption are captured in the FBLs (R1, B1, B2a and B2b) which include children’s willingness to consider FV, FV preference, outcome expectation and peer influence.

#### Social habituation (R1)

This is a self-reinforcing FBL regarding the social transmission of FV consumption behaviour from children who consume FV to those who do not.^25^ The same mechanism may exist between child-parents^26^ and child-teachers,^27^ with varying degrees of strength depending on the children’s age. However, parents’ and teachers’ role modelling has not been a part of the Scheme’s main components.^7^ FV consumption gradually enters children’s consideration as a result of educational measures^17^ and observing peers who consume FV or express their opinions about it.^25^ When children’s willingness to consider FV reaches a certain point, the social habituation loop (R1) becomes dominant, implying that more children consume FV and more social transmission takes place. If the Scheme does not raise the children’s willingness to a self-sustaining level, FV consumption will not become a social habit; this is because R1 is a regenerative path-dependent mechanism, implying that the system will take a path towards lower FV consumption, leading to non-FV eaters becoming the majority.^28^ Hence, it necessitates even more effort from the Scheme to reverse the direction of the loop.

#### Retention (B1)

Transforming information learned through educational measures and peer influence into memory codes and remembering them is a key aspect of observational learning.^29^ Rehearsing observed FV consumption behaviour over time can help stabilize it,^29^ whereas limited experience with FV can result in information loss and forgetting.^30^

#### No more unhealthy food (B2a) and children get bored of FV (B2b)

These mechanisms account for the delays between eating behaviour (FV or unhealthy food consumption) and perceived health status. The distal health outcome of FV consumption is insufficient to motivate children to choose FV over unhealthy food, which is immediately rewarding to the taste buds.^31^ Children are motivated by taste and variety,^26^ and in some cases, they get bored of FV consumption. Consequently, the influence of health educational messages on children’s eating habits falls short of their intended objectives.^32^

### Opportunity

Individuals can sustain healthy eating behaviours when opportunities are plentiful.^33^ In this section, FV availability at school and at home will be discussed in the context of the FBLs; B3a, B3b, B4, B5 and R2, which are depicted in figure 2.

#### Apples again? (B3a) and what about local producers? (B3b)

Offering a diverse selection of FV is a promising success factor for the Scheme,^7^ as it influences children’s FV preferences and may result in increased FV consumption.^26^^,^^27^ The FBLs B3a and B3b illustrate a situation in which two competing goals, the children’s desired FV variety and the Scheme’s goal of promoting local FV products, pull the system in opposite directions and undermine the Scheme’s main objective by offering a limited FV variety.^7^

#### Scheme reach at the price of its intensity (B4)

This is a balancing FBL where the Scheme coverage goal and the FV distribution’s intensity can constrain each other. ‘Coverage’ is defined as the percentage of children per country. The intensity of FV distribution is defined as the duration of implementation in weeks per school year and the frequency of FV delivery per week. Given the Scheme’s predetermined and limited financial resources, the Scheme coverage decreases as the intensity (i.e. frequency or duration) of the FV distribution increases, implying that fewer children can participate in the Scheme. Policymakers have been known to reduce the intensity of the FV distribution to enrol more children in the Scheme.^7^

#### Are teachers willing to participate in the Scheme? (R2)

If financial resources are adequate, a lack of schools’ willingness to participate in the Scheme may limit the Scheme’s coverage. Teachers’ interest and positive view of the Scheme’s benefits motivate schools to join the Scheme,^34^ thereby expanding the Scheme’s coverage. As a result of increased access to the Scheme, children’s FV consumption increases, further supporting the teacher’s positive view (R2).

#### Substitution effect (B5)

The Scheme may relieve parents of the responsibility of providing FV to their children.^34^ During the Scheme’s implementation period, children either do not bring FV from home or consume less at home because it is provided at school, reflecting the dilemma of the substitution effect. The Scheme’s effort to improve the FV provision is mitigated by balancing FBL B5. Because parents might be unaware of the recommended level of FV consumption, FV provision through the Scheme reduces parental FV provision. It should be noted that children’s picky eating,^35^ the cost, availability^35^ and time required to prepare FV^36^ may be other limiting factors for parents’ FV provision, but this is beyond the boundaries of this CLD.

### Capability

In this section, we will look at self-efficacy, self-regulation and children asking their parents for FV as capability mechanisms in the context of the FBLs; R3a, R3b, B6a and B6b, which are depicted in figure 3.

#### I can eat FV if my friend can (R3a)

R3a is a self-reinforcing FBL in which children’s self-efficacy increases as a result of their participation in the Scheme. Peer influence and educational measures aimed at equipping children with practical skills for preparing FV are among the Scheme’s activities that may serve as multipliers for self-efficacy. Positive self-efficacy has been found to predict children’s FV consumption.^37^

#### I have plenty of FV, so I may ask for more (R3b)

Perceived FV opportunities increase children’s self-efficacy and motivate them to exert pressure at home by requesting FV, leading to higher FV availability at home, which may further improve their perceived FV opportunities and self-efficacy.^38^

#### Am I eating enough FV (B6a)? and If I cannot get FV, I will forget about it (B6b)

B6a and B6b are two balancing FBLs that reflect the children’s perceived barriers to FV consumption.^37^ When a child’s desire for FV consumption, which may be influenced by the Scheme and peers, conflicts with their current FV consumption, there are two coping mechanisms, B6a and B6b. B6a causes children to ask for FV from their parents, as children are not direct agents of their environment, and B6b causes them to lower their desired FV consumption to reduce the dissatisfaction associated with not reaching their goal.^33^

## Discussion

The aim of this study was to develop a CLD that visualizes the various and interconnected mechanisms leading to the Scheme’s long-term impact on children’s FV consumption. The Scheme is implemented within a complex system; as such, utilizing the systems thinking approach enabled us to keep track of the complexity and consider how to make the different parts of the system support the aim of the Scheme.

Our CLD reflects the principle of ‘limit to growth’, a systems archetype that emphasizes that every growth path (e.g. increasing children’s FV consumption) has inherent limits. In our findings, a set of self-reinforcing FBLs related to motivation (R1), and capability (R3a and R3b) feedback on themselves and initially accelerate children’s FV consumption. However, the growth in FV consumption gradually slows down to a halt. This slowing occurs because a set of balancing FBLs with alternative goals (B4, B5, B3a and B3b), related to opportunity mechanisms, reaches a limit. Growth can be limited by resource constraints, such as insufficient financial resources or teachers’ motivation, or external responses to growth, such as parents’ reaction to the Scheme. To achieve a long-term impact on children’s FV consumption, the sources of limits within the balancing FBLs must be identified as outlined in the Results section and weakened by strengthening certain components of the Scheme as outlined below.

Financial resources, widespread awareness of recommended FV consumption, the availability of a diverse range of FV products, and school acceptance of the scheme appear to have the potential to eliminate constraints in opportunity mechanisms. While increasing financial resources is a simple solution that may ensure the Scheme is delivered with sufficient intensity and coverage, research has shown that even when financial resources are doubled, coverage remains constant while duration doubles.^39^ Thus, alternative ways to deliver the Scheme should be considered; for instance, some member states implemented the periodic rotation of the Scheme so that more schools are covered, or targeted delivery of the Scheme differentiated by SES or age.

Training teachers and letting them be role models by eating FV could help teachers buy in and encourage more children to eat FV. By including a parental component, widespread awareness of recommended FV consumption can be achieved. Active parental involvement has proven to be an effective strategy in raising awareness about FV consumption recommendations and achieving sustainable change through school-based FV programmes.^32^ However, parents’ and teachers’ role modelling has not been a part of the Scheme’s main components. The Scheme’s most widely used strategy for informing parents has been passive (i.e. newsletters), despite evidence that this is not very effective.^32^ Both schools and parents agree that parents should be more actively involved, but the Scheme lacks a clear strategy for this.^8^^,^^11^

While FBLs B3a and B3b are opportunity mechanisms affecting FV variety, they also have the potential to increase motivation, i.e. FV preferences.^26^ The Scheme has delegated FV variety decisions to schools.^7^ According to the 5-year strategy documents, some countries, particularly regarding vegetables, continue to provide a limited variety.^8^ Hence, it is recommended to maximize variety within the local context/seasonality and to supplement with occasional imported FV.

Once the limiting factors of the balancing FBLs are addressed, the self-reinforcing processes can be strengthened. In the self-reinforcing FBLs, the children’s social interaction (R1), self-efficacy (R3a) and asking for FV from parents (R3b) are among the activities that can be better utilized. As previously stated, the social transmission of FV consumption behaviour is critical for establishing FV consumption as a social norm.^25^ The Scheme should include clear strategies to transform peer pressure against FV consumption into peer support.^26^ Currently, some countries use peer role modellings^15^ to improve the group experience of consuming FV.^34^ To the best of our knowledge, there has been no study of the Scheme’s effect on children’s FV self-efficacy. However, two studies indicate that following the Scheme, children request more fruit but not vegetables at home.^8^^,^^11^ Yet, we cannot be sure that all children will request FV at home; thus, increased parental awareness is necessary. While parents play an important role in FV availability and accessibility at home, their perceptions regarding barriers to FV feeding children can influence their support.^35^ With this information in mind, focused age-appropriate educational measures can reinforce FBLs R3a and R3b so that children’s asking behaviour for FV from their parents and their FV self-efficacy work in favour.^32^

The strength of taking a systems approach is that it allowed us to map out the implicit goals of the Scheme’s key agents (e.g. children, parents, teachers and policymakers) and provide an integrated perspective on the mechanisms that shape the Scheme’s impact. The limitation of this study is that to build the CLD, we used secondary data sources such as peer-reviewed literature and official documents. The literature was not systematically reviewed, and the current and local relevance of all the evidence for different socio-demographic groups is not known. However, we reduced the risk of bias by seeking expert opinions on the CLD. Furthermore, we made a choice to focus on the goal of long-term impact on children’s FV consumption and only alluded to the goal of stabilizing the EU FV market in relation to this. The goal of obesity reduction was defined as outside the boundaries of the CLD to keep it comprehensible. The wider social system and synergies with other FV interventions, which may require various degrees of individual agency, were also defined as being outside the boundaries but have been addressed by others.^40^ This study identified feedback processes that play a significant role in determining the Scheme’s potential impact on children’s long-term FV consumption. The variables and their relationships demonstrated in these processes are well known in the literature. Nonetheless, when taken together, they provide a new, more concise perspective that allows stakeholders to see the Scheme in a broader context with an inclusive boundary of its impact on children’s FV consumption. The CLD can be a useful tool for policy discussions about the execution and impact of the Scheme to find more mutually beneficial ways of achieving the goals of children, parents, teachers and policymakers. More research on the CLDs' applicability in different contexts and socio-demographic groups should be conducted, with the participation of relevant stakeholders.

## Supplementary data

Supplementary data are available at *EURPUB* online.

## Funding

Main funding provided by Throne Holst Nutrition Research Foundation, University of Oslo, Norway with supplementary funds from the Research Council of Norway (grant number: 297894/H10). The PEN project (www.jpi-pen.eu) is funded by the Joint Programming Initiative ‘A Healthy Diet for a Healthy Life’ (JPI HDHL), a research and innovation initiative of EU member states and associated countries. The funding agencies supporting this work are (in alphabetical order of participating countries): France: Institut National de la Recherche Agronomique (INRA); Germany: Federal Ministry of Education and Research (BMBF); Ireland: Health Research Board (HRB); Italy: Ministry of Education, University and Research (MIUR); The Netherlands: The Netherlands Organisation for Health Research and Development (ZonMw); New Zealand: The University of Auckland, School of Population Health; Norway: The Research Council of Norway (RCN); Poland: The National Centre for Research and Development (NCBR).

*Conflicts of interest*: None declared.

Key points

- Factors influencing children’s long-term FV consumption and the effectiveness of school-based FV policies are interrelated.
- The interrelated motivation, opportunity and capability mechanisms highlight the crucial role of multi-component interventions in addressing children's low FV consumption.
- Ensuring sustained opportunities for children to consume FV while strengthening motivation and capability mechanisms should be the main priority in school-based FV policy programmes.

## Supplementary Material

ckac054_Supplementary_Data
