# Supplementary material for: Applying a systems perspective to understand the mechanisms of the European School Fruit and Vegetable Scheme
Source: Eur J Public Health. 2022 Nov 29;32(Suppl 4):iv107–13. doi: 10.1093/eurpub/ckac054 (PMC9706111; doi:10.1093/eurpub/ckac054)
Supplement: ckac054_Supplementary_Data [file ckac054_supplementary_data.zip › ckac054_Supplementary_Data/Zolfaghari_EU fruit Scheme_supplementary-1-literature list.docx]

**Supplementary material file 1 for: “Applying a systems’ perspective to understand the mechanisms of the European School Fruit and Vegetables Scheme”**

Author(s): Mahshid Zolfaghari, Biljana Meshkovska, Anna Banik, Carlijn B.M. Kamphuis, Birgit Kopainsky, Aleksandra Luszczynska, Celine Murrin, Nanna Lien; on behalf of the PEN consortium

Table 1 represents a full list of literature used in developing the final causal loop diagram.

| Item | Reference | Feedback loop |
| --- | --- | --- |
| 1 | Lehto E, Ray C, Haukkala A, Yngve A, Thorsdottir I, Roos E. Do descriptive norms related to parents and friends predict fruit and vegetable intake similarly among 11-year-old girls and boys? Br J Nutr. 2016;115(1):168-75. | Social habituation (R1) |
| 2 | Birch LL. Development of food preferences. Annu Rev Nutr. 1999;19:41-62. | Social habituation (R1) |
| 3 | Lowe CF, Horne PJ, Tapper K, Bowdery M, Egerton C. Effects of a peer modelling and rewards-based intervention to increase fruit and vegetable consumption in children. Eur J Clin Nutr. 2004;58(3):510-22. | Social habituation (R1) |
| 4 | Struben J, Chan D, Dubé L. Policy insights from the nutritional food market transformation model: the case of obesity prevention. Ann N Y Acad Sci. 2014;1331(1):57-75. | Social habituation (R1), Retention (B1), Substitution effect (B5) |
| 5 | Roccaldo R, Censi L, D'Addezio L, Berni Canani S, Gennaro L. A teachers’ training program accompanying the “School Fruit Scheme” fruit distribution improves children’s adherence to the Mediterranean diet: an Italian trial. Int J Food Sci Nutr. 2017;68(7):887-900. | Social habituation (R1) |
| 6 | Rasmussen M, Krølner R, Klepp K-I, Lytle L, Brug J, Bere E, et al. Determinants of fruit and vegetable consumption among children and adolescents: a review of the literature. Part I: quantitative studies. Int J Behav Nutr Phys Act. 2006;3(1):22. | Social habituation (R1), I can eat FV if my friend can (R3a), Am I eating enough FV (B6a)? and If I can’t get FV I’ll forget about it (B6b) |
| 7 | Ismail MR, Seabrook JA, Gilliland JA. Process evaluation of fruit and vegetables distribution interventions in school-based settings: A systematic review. Prev Med Rep. 2021;21:101281. | Social habituation (R1) |
| 8 | Krolner R, Rasmussen M, Brug J, Klepp KI, Wind M, Due P. Determinants of fruit and vegetable consumption among children and adolescents: a review of the literature. Part II: qualitative studies. Int J Behav Nutr Phys Act. 2011;8. | Social habituation (R1), No more unhealthy food (B2a) and children get bored of FV (B2b), Apples again? (B3a) and what about local producers? (B3b) |
| 9 | Reynolds KD, Hinton AW, Shewchuk RM, Hickey CA. Social cognitive model of fruit and vegetable consumption in elementary school children. J Nutr Educ. 1999;31(1):23-30. | Social habituation (R1) |
| 10 | Brug J, Tak NI, Velde SJT, Bere E, de Bourdeaudhuij I. Taste preferences, liking and other factors related to fruit and vegetable intakes among schoolchildren: results from observational studies. Br J Nutr. 2008;99:S7-S14. | Social habituation (R1) |
| 11 | Pearson N, Biddle SJ, Gorely T. Family correlates of fruit and vegetable consumption in children and adolescents: a systematic review. Public Health Nutr. 2009;12(2):267-83. | Social habituation (R1), Substitution effect (B5), |
| 12 | Van der Horst K, Oenema A, Ferreira I, Wendel-Vos W, Giskes K, van Lenthe F, et al. A systematic review of environmental correlates of obesity-related dietary behaviors in youth. Health Educ Res. 2007;22(2):203-26. | Social habituation (R1) |
| 13 | Birch LL, Gunder L, Grimm-Thomas K, Laing DG. Infants' consumption of a new food enhances acceptance of similar foods. Appetite. 1998;30(3):283-95. | Social habituation (R1) |
| 14 | Haß J, Lischetzke T, Hartmann M. Does the distribution frequency matter? A subgroup specific analysis of the effectiveness of the EU School Fruit and Vegetable Scheme in Germany comparing twice and thrice weekly deliveries. Public Health Nutr. 2018;21(7):1375-87. | Social habituation (R1) |
| 15 | Kwasnicka D, Dombrowski SU, White M, Sniehotta F. Theoretical explanations for maintenance of behaviour change: a systematic review of behaviour theories. Health Psychol Rev. 2016;10(3):277-96. | No more unhealthy food (B2a) and children get bored of FV (B2b), Am I eating enough FV (B6a)? and If I can’t get FV I’ll forget about it (B6b) |
| 16 | Mullan B, Novoradovskaya E. Habit mechanisms and behavioural complexity. The psychology of habit: Springer; 2018. p. 71-90. | No more unhealthy food (B2a) and children get bored of FV (B2b) |
| 17 | Gardner B, Rebar AL. Habit formation and behavior change. Oxford Research Encyclopedia of Psychology2019. | No more unhealthy food (B2a) and children get bored of FV (B2b) |
| 18 | Laureati M, Bergamaschi V, Pagliarini E. School-based intervention with children. Peer-modeling, reward and repeated exposure reduce food neophobia and increase liking of fruits and vegetables. Appetite. 2014;83:26-32. | Apples again? (B3a) and what about local producers? (B3b) |
| 19 | Staudigel M, Lingl C, Roosen J. Preferences versus the Environment: How Do School Fruit and Vegetable Programs Affect Children's Fresh Produce Consumption? Applied economic perspectives and policy. 2019;41(4):742-63. | No more unhealthy food (B2a) and children get bored of FV (B2b) |
| 20 | He MZ, Beynon CE, Gritke JL, Henderson ML, Kurtz JM, Bouck MS, et al. Children's Perceptions of the Northern Fruit and Vegetable Program in Ontario, Canada. J Nutr Educ Behav. 2012;44(6):592-6. | Apples again? (B3a) and what about local producers? (B3b) |
| 21 | Aarestrup AK, Krolner R, Jorgensen TS, Evans A, Due P, Tjornhoj-Thomsen T. Implementing a free school-based fruit and vegetable programme: barriers and facilitators experienced by pupils, teachers and produce suppliers in the Boost study. BMC Public Health. 2014;14. | Apples again? (B3a) and what about local producers? (B3b) |
| 22 | Evaluation Study School Fruit Campaign (2014-2016) Belgium 2016. | Apples again? (B3a) and what about local producers? (B3b), Are teachers willing to participate in the Scheme? (R2), Substitution effect (B5) |
| 23 | Food Dudes Evaluation 2016. Ireland: National Nutrition Surveillance Centre, UCD; Real Nation; Bord Bía; Department of Agriculture, Food and the Marine; 2016. | Apples again? (B3a) and what about local producers? (B3b), Are teachers willing to participate in the Scheme? (R2) |
| 24 | Results evaluation EU School Fruit Programme 2011-2012 / 2015-2016. In: Ministerie van Economische Zaken, editor. The Netherlands 2017. | Apples again? (B3a) and what about local producers? (B3b), Are teachers willing to participate in the Scheme? (R2) |
| 25 | Seper MK. Evaluation of School Fruit and Vegtebales Program school year 2015/2016. Austria: Austrian Agency for Health and Food Safety GmbH, Integrative risk assessment, data and statistics, Center for Nutrition & Prevention; 2016. | Apples again? (B3a) and what about local producers? (B3b) |
| 26 | Elles A, Kliebisch C, Becker A, Altmann M, Stenger M. Evaluation of the European School Fruit Scheme. Bonn, Luxembourg: AFC Management Consulting AG, CO CONCEPT Marketing Consultng, Directorate-General for Agriculture and Rural Development (European Commission); 2012. Report No.: AGRI-2011-EVAL-06. | Apples again? (B3a) and what about local producers? (B3b),  Scheme reach at the price of its intensity (B4) |
| 27 | Summary of the evaluation results of the Federal Republic of Germany participating in the EU School Fruit and Vegetable Program (SOGP) for the school years 2011/2012 - 2015/2016. Germany: Federal Ministry of Food and Agriculture; 2016. | Are teachers willing to participate in the Scheme? (R2), Substitution effect (B5) |
| 28 | Jørgensen TS, Krølner R, Aarestrup AK, Tjørnhøj-Thomsen T, Due P, Rasmussen M. Barriers and facilitators for teachers' implementation of the curricular component of the boost intervention targeting adolescents' fruit and vegetable intake. J Nutr Educ Behav. 2014;46(5):e1-e8. | Are teachers willing to participate in the Scheme? (R2) |
| 29 | Potter SC, Schneider D, Coyle KK, May G, Robin L, Seymour J. What works? Process evaluation of a school‐based fruit and vegetable distribution program in Mississippi. J Sch Health. 2011;81(4):202-11. | Are teachers willing to participate in the Scheme? (R2) |
| 30 | Maschkowski G, Hartmann M, Grebitus C. Analyzing Parental Influence on Fruit and Vegetable Consumption. 2010. | Substitution effect (B5) |
| 31 | Gerritsen S, Renker-Darby A, Harré S, Rees D, Raroa DA, Eickstaedt M, et al. Improving low fruit and vegetable intake in children: Findings from a system dynamics, community group model building study. PLoS One. 2019;14(8):e0221107. | Substitution effect (B5) |
| 32 | Slater A, Bowen J, Corsini N, Gardner C, Golley R, Noakes M. Understanding parent concerns about children’s diet, activity and weight status: an important step towards effective obesity prevention interventions. Public Health Nutr. 2010;13(8):1221-8. | Substitution effect (B5) |
| 33 | DeSmet A, Liu Y, De Bourdeaudhuij I, Baranowski T, Thompson D. The effectiveness of asking behaviors among 9-11 year-old children in increasing home availability and children's intake of fruit and vegetables: results from the Squire's Quest II self-regulation game intervention. Int J Behav Nutr Phys Act. 2017;14(1):51. | Substitution effect (B5) |
| 34 | Gaines A, Turner LW. Improving Fruit and Vegetable Intake Among Children. Californian Journal of Health Promotion. 2009;7(1):52-66. | I can eat FV if my friend can (R3a), Am I eating enough FV (B6a)? and If I can’t get FV I’ll forget about it (B6b) |
| 35 | De Bourdeaudhuij I, Velde ST, Brug J, Due P, Wind M, Sandvik C, et al. Personal, social and environmental predictors of daily fruit and vegetable intake in 11-year-old children in nine European countries. Eur J Clin Nutr. 2008;62(7):834-41. | I can eat FV if my friend can (R3a). |
| 36 | Geller KS, Dzewaltowski DA, Rosenkranz RR, Karteroliotis K. Measuring children’s self‐efficacy and proxy efficacy related to fruit and vegetable consumption. J Sch Health. 2009;79(2):51-7. | I have plenty of FV, so I may ask for more (R3b) |
| 37 | Brug J. Determinants of healthy eating: motivation, abilities and environmental opportunities. Fam Pract. 2008;25 Suppl 1:i50-5. | Am I eating enough FV (B6a)? and If I can’t get FV I’ll forget about it (B6b) |
| 38 | Bandura A. Social-learning theory of identificatory processes. Handbook of socialization theory and research. 1969;213:262. | Retention­ (B1) |
| 39 | Gerritsen S, Harré S, Swinburn B, Rees D, Renker-Darby A, Bartos AE, et al. Systemic barriers and equitable interventions to improve vegetable and fruit intake in children: interviews with national food system actors. Int J Environ Res Public Health. 2019;16(8):1387. | Social habituation loop (R1), I have plenty of FV, so I may ask for more (R3b) |
