# Supplementary material for: Applying a systems perspective to understand the mechanisms of the European School Fruit and Vegetable Scheme
Source: Eur J Public Health. 2022 Nov 29;32(Suppl 4):iv107–13. doi: 10.1093/eurpub/ckac054 (PMC9706111; doi:10.1093/eurpub/ckac054)
Supplement: ckac054_Supplementary_Data [file ckac054_supplementary_data.zip › ckac054_Supplementary_Data/Zolfaghari_EU fruit Scheme_supplementary-2-analysis example.docx]

**Supplementary material file 2 for: “Applying a systems’ perspective to understand the mechanisms of the European School Fruit and Vegetables Scheme”**

Author(s): Mahshid Zolfaghari, Biljana Meshkovska, Anna Banik, Carlijn B.M. Kamphuis, Birgit Kopainsky, Aleksandra Luszczynska, Celine Murrin, Nanna Lien; on behalf of the PEN consortium

The table below depicts an exemplary coding procedure from step 1 to 3, which resulted in the self-reinforcing loop of social habituation (R1).

(In the table FV and F & V stands for fruits and vegetables).

| Output of step 1 (a list of exemplary data segments) | Information source | Output of step 2 (coding chart) | Output of step 3 (simple words-and-arrow diagrams) |
| --- | --- | --- | --- |
| We noted that already among children in the elementary school friends seem to have an impact on each other’s FV intake. The strong impact of best friends implies that children’s whole social environment, not only the family context, should be taken into account when aiming at increasing children’s FV intake. | (1) | Cause: peer influence  Effect: FV consumption  Relationship type: positive | Peer influence 🡪 FV consumption |
| Children’s preferences for and consumption of [initially] disliked vegetables were enhanced when children had opportunities to observe peers selecting and eating foods that the observing child disliked. | (2) | Cause: observing peers  Effect: children’s vegetable preferences, children’s vegetable consumption  Relationship type: positive | Peer influence 🡪 children’s FV preference, children’s FV consumption |
| The peer modelling and rewards-based intervention was shown to be effective in bringing about substantial increases in children’s consumption of, and expressed liking for, fruit and vegetables. | (3) | Cause: peer modelling  Effect: children’s FV consumption, and expressed liking  Relationship type: positive | Peer influence🡪 children’s FV consumption, and FV liking |
| Consumers consider a [food] category only when sufficiently familiar with it. Constrained by limited information-processing capacity and aspirations to objectively evaluate all options, consumer attention is guided by different channels of influence: by firms’ marketing (efforts to shape the category and brands); by social exposure (word of mouth, media attention, social norms, and peer pressure); and by individual habituation, sensitization, and brand loyalty. | (4) | Cause: Social exposure  Effect: consideration of a food category  Relationship type: positive | Social exposure🡪 consideration of a food category |
| Category market share [of a food category] is a function of the utility that consumers derive from related products, but also of the population-level propensity to consider (PtC) the category. | (4) | Cause: the population-level propensity to consider the category  Effect: food category market share  Relationship type: positive | the population-level propensity to consider a food category🡪 food category market share |
| As propensity to consider [a food category] builds, consumption of the category grows, providing further [social] exposure and increase in propensity to consider (reinforcing feedback R1, social exposure) | (4) | Cause: consumption of the food category, propensity to consider [a food category]  Effect: social exposure  Type of relationship: positive | the population-level propensity to consider a food category🡪 Consumption of a food category🡪 social exposure |
| Category market share is a function of the utility that consumers derive from related products.  Market share of category c by firms f within population segment d depends on consumers’ relative affinity with category-related products. | (4) | Cause: consumers affinity with food category, product utility  Effect: category market share  Type of relationship: positive | product utility 🡪consumers affinity with food category🡪 category market share |
| The teachers who delivered the intervention in their classes, using the TP [teaching pack] developed within the Italian School Fruit Scheme Accompanying Measures, mentioned the enthusiasm, high participation, and involvement of the children in the activities, and more willingness to try and eat FV, both at school and at home. | (5) | Cause: educational measures  Effect: willingness to try and eat FV  Relationship type: positive | educational measure🡪 children’s willingness to try and eat FV |
| Preference is the personal factor that has been examined most extensively. Eleven papers analysed the influence of preferences, and in all 11 papers a positive association between preferences and children and adolescents' intake of fruit and/or vegetables was observed. | (6) | Cause: preferences  Effect: Children’s FV consumption  Type of relationship: positive | Preferences 🡪 Children’s FV consumption |
| studies highlighted the benefits of […] variety of served FVs (Bouck et al., 2011; Potter et al., 2011), which were all positively associated with children’s degree of liking/enjoyment, and ultimately, their consumption of FVs. | (7) | Cause: Variety  Effect: Children’s liking/enjoyment, FV consumption  Type of relationship: positive | Variety🡪 Children’s liking/enjoyment 🡪 FV consumption |
| Variety and choice,  Children from the US and New Zealand reported they would eat more fruit and vegetables if school offered a larger variety that matched their preferences and served them fresh. Some children said that the choice of fruit in school was limited to canned fruit salad which they did not like […]. | (8) | Cause: variety  Effect: preferences  Type of relationship: positive | Variety🡪 preferences |
| Among environmental constructs, availability of F&V is necessary for exposure to occur, leading to increased preferences and increased consumption. | (9) | Cause: availability of F&V  Effect: preferences, consumption  Type of relationship: positive | availability of FV 🡪 FV preferences, FV consumption |
| For example, while consumers are sensitive to economic stimuli that alter food cost and convenience, the actual change of food consumption patterns is conditioned by social influence and habits, as well as by food availability, all factors being intricately linked. | (4) | Cause: food availability  Effect: food consumption  Type of relationship: positive | food availability 🡪 food consumption |
| Availability of fruit and vegetables at home emerged as an important factor affecting fruit and vegetable consumption in 15 studies from different countries.  Availability and exposure to fruit and vegetables in school, Irrespective of country setting, it is a consistent finding across most studies that fruit and vegetables are only available in small quantities in school or not available at all. | (8) | Cause: availability of fruit and vegetable at home, availability and exposure to fruit and vegetables in school  Effect: FV consumption  Type of relationship: positive | availability of fruit and vegetable at home, Availability and exposure to fruit and vegetables in school 🡪 FV consumption |
| These results reveal that minimal experience with a new food enhances the infants ‘acceptance not only of that food but also of other, similar foods. ‘‘Similar’’ food in this context means other pureed fruits for infants given repeated feedings of bananas, or other pureed vegetables for infants repeatedly fed pureed peas. | (10) | Cause: repeated experience with food  Effect: food acceptance  Type of relationship: positive | FV consumption 🡪 willingness to consider FV |

Step 4: The aim of this step was to generalise the output of step 3 with the use of axial coding and causal maps. Its output is presented in Figure 1. It is worth noting that in the axial coding process, we consider two processes for learning preferences: innate and acquired. The enjoyment variable captures innate fruits and vegetable preferences. The willingness to consider fruits and vegetables captures acquired preferences that reflect social and physical exposure to fruits and vegetables.

Step 5: We kept a record of the first two columns of the above table to have an overview of all the data sources.

Figure 1– Causal loop diagram (CLD) representing the social habituation loop influencing children’s fruit and vegetable consumption. A CLD visualises the interconnections among variables as well as feedback loops (FBL). For each arrow, the polarity indicates whether variables move in the same (positive sign) or opposite (negative sign) directions (all else being constant). There are two types of FBLs in a CLD: balancing FBL (B), which oppose change introduced to the system, and reinforcing FBL (R), which amplify change introduced to the system. The constructs presented in the boxes in Figure 1 illustrate the variables that were in various data segments, representing the same variables in the final causal loop diagram.

References

1. Lehto E, Ray C, Haukkala A, Yngve A, Thorsdottir I, Roos E. Do descriptive norms related to parents and friends predict fruit and vegetable intake similarly among 11-year-old girls and boys? Br J Nutr. 2016;115(1):168-75.

2. Birch LL. Development of food preferences. Annu Rev Nutr. 1999;19:41-62.

3. Lowe CF, Horne PJ, Tapper K, Bowdery M, Egerton C. Effects of a peer modelling and rewards-based intervention to increase fruit and vegetable consumption in children. Eur J Clin Nutr. 2004;58(3):510-22.

4. Struben J, Chan D, Dubé L. Policy insights from the nutritional food market transformation model: the case of obesity prevention. Ann N Y Acad Sci. 2014;1331(1):57-75.

5. Roccaldo R, Censi L, D'Addezio L, Berni Canani S, Gennaro L. A teachers’ training program accompanying the “School Fruit Scheme” fruit distribution improves children’s adherence to the Mediterranean diet: an Italian trial. Int J Food Sci Nutr. 2017;68(7):887-900.

6. Rasmussen M, Krølner R, Klepp K-I, Lytle L, Brug J, Bere E, et al. Determinants of fruit and vegetable consumption among children and adolescents: a review of the literature. Part I: quantitative studies. Int J Behav Nutr Phys Act. 2006;3(1):22.

7. Ismail MR, Seabrook JA, Gilliland JA. Process evaluation of fruit and vegetables distribution interventions in school-based settings: A systematic review. Prev Med Rep. 2021;21:101281.

8. Krolner R, Rasmussen M, Brug J, Klepp KI, Wind M, Due P. Determinants of fruit and vegetable consumption among children and adolescents: a review of the literature. Part II: qualitative studies. Int J Behav Nutr Phys Act. 2011;8.

9. Reynolds KD, Hinton AW, Shewchuk RM, Hickey CA. Social cognitive model of fruit and vegetable consumption in elementary school children. J Nutr Educ. 1999;31(1):23-30.

10. Birch LL, Gunder L, Grimm-Thomas K, Laing DG. Infants' consumption of a new food enhances acceptance of similar foods. Appetite. 1998;30(3):283-95.
