# Supplementary material for: Applying a systems perspective to understand the mechanisms of the European School Fruit and Vegetable Scheme
Source: Eur J Public Health. 2022 Nov 29;32(Suppl 4):iv107–13. doi: 10.1093/eurpub/ckac054 (PMC9706111; doi:10.1093/eurpub/ckac054)
Supplement: ckac054_Supplementary_Data [file ckac054_supplementary_data.zip › ckac054_Supplementary_Data/Zolfaghari_EU fruit Scheme_Supplementary-3-methodology rationale.docx]

**Supplementary material file 3 for: “Applying a systems’ perspective to understand the mechanisms of the European School Fruit and Vegetables Scheme”**

Author(s): Mahshid Zolfaghari, Biljana Meshkovska, Anna Banik, Carlijn B.M. Kamphuis, Birgit Kopainsky, Aleksandra Luszczynska, Celine Murrin, Nanna Lien; on behalf of the PEN consortium

**System dynamics modelling process for developing causal loop diagrams (CLD) – Rationale, problem articulation, formulation of dynamic hypothesis and the boundary of CLD**

We applied systems thinking and the conceptualisation steps from system dynamics approach^1^ and used peer-reviewed articles and official documents to identify and represent the interconnected mechanisms at work in the European School Fruit and Vegetables Scheme (the Scheme) to achieve a long-term impact on children’s fruit and vegetable (FV) consumption. The aim of a systems thinking, or system dynamics project is to model the main pathways underlying a certain problem rather than the entire system. *“The usefulness of models lies in the fact that they simplify reality, creating a representation of it we can comprehend.”^1^* It is thus important to clearly define the objective of the causal loop diagram (CLD), namely the dynamic problem that it explains, and the system structure (interconnected mechanisms) that generates the problematic behaviour. In line with the system dynamics approach, we therefore did not systematically review all the relevant literature for each mechanism in the CLD. The remainder of this document expands on the CLD's primary objective and reflects on the iterations that defined the CLD's scope and boundaries, as well as the use of the literature.

**Problem articulation**

The goal of the study from the outset was to use systems thinking approach to describe the structure of the impact of the Scheme. The consumption of fruits and vegetables by children was identified as the variable of interest for impact, and the specific problematic behaviour lies in the persistently low levels of consumption over time.

**Formulation of the dynamic hypothesis**

After characterising the problem, the next step was to develop a dynamic hypothesis, which is *“a structure that the modeller advances to explain a dynamic behaviour of interest”.*^2^ The dynamic hypothesis is open to amendment or abandonment throughout the modelling process.^1^ The remaining modelling effort was for us to validate our dynamic hypothesis, either through gathering knowledge from the literature or expert opinions. With this in mind, we drew on documents related to the Scheme, which were gathered from the European Commission’s website^3^ and the European Commission policy officer responsible for the Scheme. These documents, together with peer-reviewed studies on the Scheme that were obtained by our research group for another ongoing study on the Scheme, enabled initial comprehension of it, including its main structure and success variables such as the relative number of participating children, high frequency of distribution, a wide range of products, and free distribution.

To ensure that we build on existing evidence, we searched scholarly databases such as PubMed, Web of Science (core collection), Google Scholar, and system dynamics bibliography^4^ for previous system dynamics or systems thinking works that have a formal model or CLD that describes the structural mechanisms of any nutrition policy at the micro level; figure 1 shows an exemplary set of concepts that were searched for in this step (figure1, step 1B).

Taking the 5 key steps^5^ to analyse the literature resulted in the first draft of the CLD (figure 2). For additional information about the development of the CLD, see supplementary material 2 and the explanation in the manuscript.

The preliminary CLD (figure 2) was validated through three separate meetings with two individuals and a group of experts, following the disconfirmatory interview guidelines for presentation and discussion.^5^ The individual experts included an economist previously involved in the Scheme evaluation, a public health nutritionist who had worked on establishing school-based FV programmes, and a group of eight public health nutrition researchers with diverse disciplinary backgrounds and expertise in children’s FV consumption and school-based interventions. It should be noted that disconfirmatory interview guidelines^5^ emphasise the importance of the individual over the group for assessing the structure, which we addressed this in the latter consultation, by selecting each individual in the group to reduce the risk of one or two (dominant) individuals in the group anchoring the rest of the group's thoughts.

At these meetings, the following topics from the presented CLD were deemed important: the Scheme implementation, children's FV determinants, and habituation mechanisms.

The highlighted topics in these meetings prompted us to conduct more research in the literature to ensure we caught the mechanisms that could presumably influence both the Scheme's success variables and children's FV consumption. For a visualization of our procedure for testing our dynamic hypothesis, please refer to figure 1.

Exploring the literature was done with the purpose of obtaining an understanding of the mechanisms relevant to the aim of the study – to provide an integrated perspective of the multiple settings, agents, components, and interconnected mechanisms that are at work in this nutrition policy programme to achieve a long-term impact on children’s FV consumption and identify ways to enhance their influence. The literature search, similar to the previous step, involved searching through scholarly databases such as PubMed, Web of Science (core collection), and Google Scholar, and figure 1 shows an exemplary set of concepts that were searched (figure 1, step 4).

A single researcher (the first author) carried out this procedure and developed the CLD to ensure that the CLD could communicate a thorough understanding of the literature.^6^ However, to reduce the risk of bias, the core modelling team (M.Z., B.M., and N.L.), each with knowledge and experience in system dynamics, political science, qualitative methods, and public health nutrition, met weekly throughout the process to revise the CLD and evaluate the key variables and their causal relationships. If a mechanism or a variable needed to be investigated more, as a result of these discussions, the first author would explore the literature to find an explanation to understand how that part of the system is working.

The final CLD was presented to a group of experts with expertise in behavioural science, school based nutrition policies and system dynamics, following the disconfirmatory interview guidelines.^5^ Similar to the previous group consultation, the experts were chosen in such a way that no single person could inﬂuence the group's opinions. In addition, prior to the meeting, experts were given a written explanation of the CLD and asked to provide comments and concerns about the structure. The purpose of the group meeting was to increase consensus and agreement among the experts.

At this final meeting, a concern was raised about loop R2 that individual motivated teachers take the initiative to participate in the Scheme, and thus participation should not be defined solely on the basis of seeing children’s FV consumption. To address this, we added a variable called “teachers’ intrinsic motivation” and updated our CLD and explanation of the loop (figure 4).

**CLD boundary**

The process of developing a CLD is not linear; rather, it undergoes numerous iterations and is constantly questioned and tested. The primary purpose of the CLD established its boundary and scope, but what we learned during the process also informed the CLD's purpose and boundary.

*“Model boundary is a border enclosing the parts of system structure needed to generate the behaviour of interest.”^2^*

The second draft of the CLD (figure 3) included mechanisms related to individuals, families, schools, suppliers, and policymakers. To answer all conceivable questions about the Scheme’s impact, the CLD would have to include an overwhelming number of variables, which would compromise its usefulness and comprehensibility. As a result, even though we included school implementation and supplier mechanisms based on expert consultations, we later had to scale back our CLD. For the reasons stated, we chose to only focus on the mechanisms of impact of the Scheme (the direct provision and accompanying measures) that directly influence children and their decisions.

**References**

1. Sterman J. Business dynamics: Irwin/McGraw-Hill c2000.. 2010.

2. Ford DN. A system dynamics glossary. System dynamics review. 2019;35(4):369-79.

3. EU Commission. School scheme explained: European Commission 2020 [Available from: <https://ec.europa.eu/info/food-farming-fisheries/key-policies/common-agricultural-policy/market-measures/school-fruit-vegetables-and-milk-scheme/school-scheme-explained_en>.

4. System Dynamics Society. Biliography [Available from: <https://systemdynamics.org/bibliography/>.

5. Andersen DL, Luna‐Reyes LF, Diker VG, Black L, Rich E, Andersen DF. The disconfirmatory interview as a strategy for the assessment of system dynamics models. Syst Dyn Rev. 2012;28(3):255-75.

6. Eker S, Zimmermann N. Using Textual Data in System Dynamics Model Conceptualization. Systems. 2016;4(3).

**Figures**

**
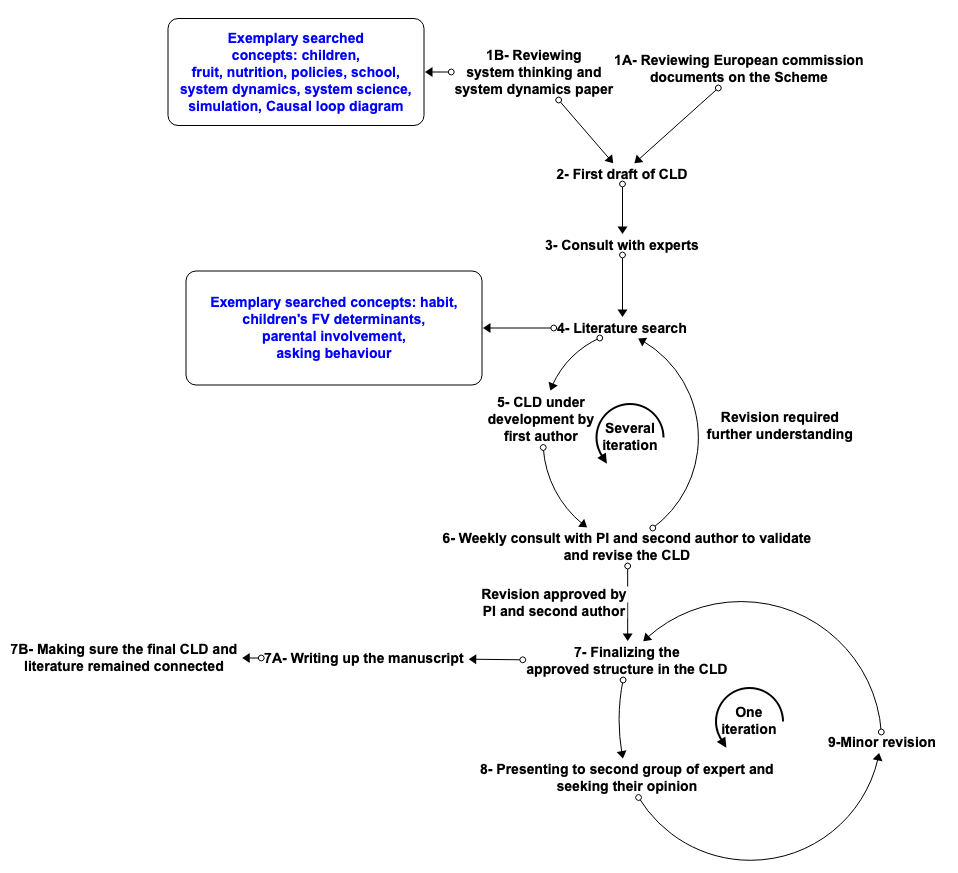
**

Figure 1- The workflow of this study is visualised to explain better our approach for testing our dynamic hypothesis.

Figure 2- The first draft of the causal loop diagram that was presented to the experts.

Figure 3- The second draft of the causal loop diagram following the third round of expert consultation. The Scheme's implementation parts were included in this causal loop diagram, which further had to be removed for causal loop diagram to remain understandable.

Figure 4­- The final causal loop diagram, which depicts the Scheme’s mechanisms of impact that directly influence children and their decisions. The red circle shows the changes made to the causal loop diagram after the final consultations with the experts.
